# Accidental Awareness Under General Anesthesia During Cesarean Section: An Observational Study

**DOI:** 10.7759/cureus.37118

**Published:** 2023-04-04

**Authors:** Suzana Sobot Novakovic, Sanja Cuk, Zoran Malusic, Dragan Sandic, Dalibor Vranjes

**Affiliations:** 1 Clinic for Anesthesiology and Intensive Care, University Clinical Center of the Republic of Srpska, Banja Luka, BIH; 2 University of Banja Luka, Department of Medicine, Banja Luka, BIH

**Keywords:** observational study, cesarean section (cs), obstetric anesthesia, general anesthesia, awareness during general anesthesia

## Abstract

Background

A Caesarean section (CS) if performed under general anesthesia (GA) is a procedure with an increased risk of accidental awareness. This study aimed to examine the incidence of accidental awareness under GA in hospitals in the Republic of Srpska (Bosnia and Herzegovina) where GA for CS is performed in a significantly higher percentage compared to spinal anesthesia.

Methodology

In the period from 2016 to 2018, a multicenter, prospective, observational study was conducted in five medical centers in the Republic of Srpska (Bosnia and Herzegovina). The study included 1,161 patients who underwent CS. A total of 427 (36.7%) patients had elective and 734 (63.3%) had emergency CSs. The patients were surveyed postoperatively using the modified Brice questionnaire.

Results

Of the 1,161 patients included in the study, 12 (1.03%) reported memory in the period between induction and emergence of anesthesia. Five (0.43%) of them reported definite and seven (0.6%) possible and unlikely awareness. Significant psychological trauma due to pain was reported by two patients. Dreams during anesthesia were reported by 42 patients (3.61%) and five of them stated that the dreams were unpleasant.

Conclusions

Accidental awareness under GA during CS has a significant incidence in medical centers in the Republic of Srpska (Bosnia and Herzegovina). According to our findings, creating new protocols for GA when performing CS is necessary.

## Introduction

Accidental awareness under general anesthesia (AAGA) is defined as the spontaneous recall of events that occurred during general anesthesia (GA). This is an extremely stressful event for the patient, which can result in serious psychological consequences [1]. The incidence of AAGA is low and usually ranges from 0.1% to 0.2% [1,2]. The risk of AAGA depends on several factors related to the patient (alcohol consumption, abuse of psychoactive substances, and hemodynamic instability), the type of surgical procedure, and the anesthesia itself [3]. AAGA appears more often in certain types of surgical procedures such as trauma surgery, craniotomy, cardiac surgery, and Caesarean section (CS) [4,5].

In CS, the main reason for the occurrence of AAGA is light anesthesia. Light anesthesia is applied to reduce the adverse effect of anesthetics on the mother and fetus due to the objective fear of respiratory depression in the newborn, uterine atony, and reduction of blood perfusion to the placenta [6,7]. AAGA during CS is one of the reasons why regional anesthesia is preferred in obstetrics. In the last few decades, this has resulted in a significant reduction in the frequency of performing GA for CS, although its use is still important in specific emergencies [8]. The reported incidence of AAGA is low. Studies investigating AAGA were usually performed on a large sample of patients [9,10]. In hospitals in the Republic of Srpska (RS) (Bosnia and Herzegovina or BIH), traditionally GA for CS is preferred over spinal anesthesia (SA). The most common reason is the refusal of patients to have this type of anesthesia for CS. Patients undergoing CS mostly have a possibility of choosing anesthesia and usually do not want to be awake during the operation due to the fear of pain and discomfort. Consequently, anesthesiologists perform SA less frequently and feel safer and more comfortable with GA for CS. This is why they have more experience with GA compared to SA, which leads to a higher percentage of GA for CS. Another issue in our hospitals is the lack of clear protocols for anesthesia for CS. We do not have imposed anesthesia standards for CS. This is one of the main reasons we decided to conduct this observational study to get a cross-section of the current situation in obstetrics and identify potential problems when it comes to the quality of service.

The primary objective of the study was to determine the incidence of AAGA in medical centers in RS (BIH). The secondary objective was to examine whether there is a need to create new protocols for anesthesia for CS in our hospitals.

## Materials and methods

A multicenter, prospective, observational study performed in the period from 2016 to 2018 included 1,161 patients who underwent GA for CS. The study was approved by the Ethics Committee of the University Clinical Center of the Republic of Srpska (UCC RS) (No. 01-9-670.2\16) and conducted in five medical centers that accepted participation in this research.

The study included all patients aged over 18 years with the American Society of Anesthesiologists (ASA) physical statuses 2 and 3 who gave birth via CS. Exclusion criteria were as follows: psychological condition and state of consciousness that made it impossible for the patients to have a conversation and fill in the questionnaire; patients' refusal to participate; patients with inadequately completed questionnaires; patients who subsequently requested exclusion from the study; death of the newborn; or all patients with significant perioperative bleeding.

The patients underwent GA that was not standardized by the study protocol. Propofol or thiopental was used for induction of anesthesia. Anesthesia was maintained with sevoflurane. Intubation relaxation was achieved with succinylcholine and maintained with pancuronium or atracurium. Intraoperative analgesia was maintained with fentanyl.

Each hospital had a researcher assigned to coordinate data collection. To determine the existence of AAGA and dreams the modified Brice questionnaire was used [11]. Basic demographic data were taken for each patient. The questionnaire contained six questions with suggested answers:

1. What is the last thing you remember before going to sleep?

2. What is the first thing you remember after waking up?

3. Do you remember anything between going to sleep and waking up?

4. Did you have dreams during the procedure?

5. Did your dreams disturb you?

6. What was the worst thing about your surgical procedure?

The same questionnaire was used in the British National Study [11], and after translation and adaptation, it was applied in this study. The questionnaire contained multiple-choice questions (MCQs) with the possibility of adding personal experiences not included in the offered answers.

Depending on the conditions in certain hospitals, patients were interviewed by the researcher who filled in the questionnaires while talking to the patients or the patients were given the questionnaire and filled them in themselves without talking to the researcher. The patients were interviewed or filled in the questionnaires 24 hours after the surgical procedure. Those who reported events that could indicate AAGA or dreaming were interviewed or filled in the questionnaire again, 72 hours after the first interview.

Depending on their answers, patients were categorized into three groups: definite awareness, possible and unlikely awareness, and no awareness. Additionally, patients with suspected AAGA were classified into five classes according to the Michigan Awareness Classification Instrument (MACI) 2: Class 0, no awareness; Class 1, isolated auditory sensations; Class 2, tactile sensations (e.g. surgical stimulation or endotracheal tube); Class 3, pain; Class 4, paralysis (feeling unable to move, speak, or breathe); and Class 5, paralysis and pain. A special *D* designation added to each of these classes indicates distress for all patients who reported feelings of fear, anxiety, suffocation, feeling like they were going to die, or other similar descriptions.

In the case of reporting AAGA, a clinical psychologist visited the patients to identify the existence of psychological trauma and the need for additional evaluation after leaving the hospital.

At the end of the study, researchers created a survey with 13 questions related to anesthesia management techniques and the use of anesthetics for GA during CS. The survey was created online and forwarded via e-mail to anesthesiologists and anesthesia residents who performed GA for CS during the study period.

Sample size

The minimum reported incidence of AAGA in earlier studies was 0.1% [1,2]; therefore, a minimum sample size of 1,000 patients was necessary for this study.

Statistical analysis

IBM Corp. Released 2011. IBM SPSS Statistics for Windows (Version 20.0, IBM Corp., Armonk, NY, USA) was used for data processing. The results are presented using tables and graphs. Categorical data are presented by absolute and relative frequencies. Numerical data are described by arithmetic mean and standard deviation.

## Results

**Figure 1:**
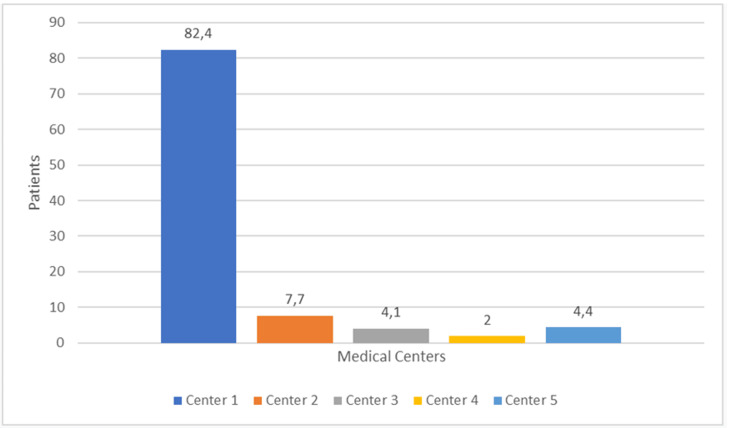
Sample structure according to the institution where the delivery took place.

**Figure 2:**
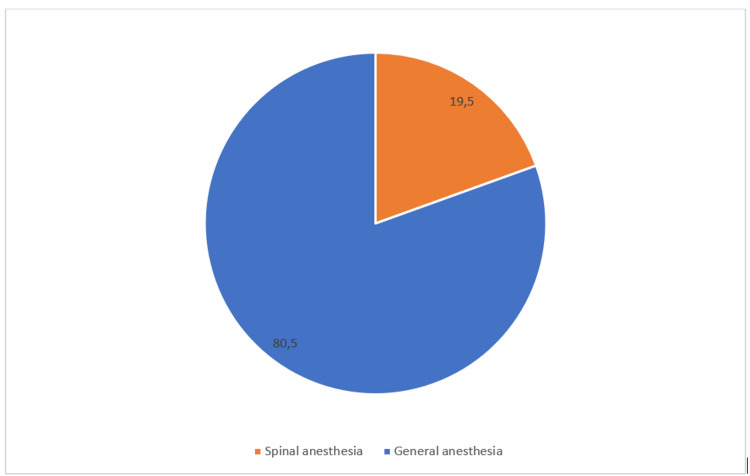
Percentage of general and spinal anesthesia for Caesarean section in medical centers in RS (BIH). RS, Republic of Srpska; BIH, Bosnia and Herzegovina

During 16 months, the study included a total of 1,236 patients, of whom 1,161 (93.5%) met the conditions for participation in the research. A total of 75 (6.6%) patients were subsequently excluded from the study. Age, educational profile, ASA status, reasons for exclusion, and type of surgery based on the urgency of all patients and those with definite awareness are listed in Table 1. The sample structure according to the institution where the delivery took place is shown in Figure 1. During the study period, 1,161 (80%) patients underwent CS under GA, and 282 of them (20%) received spinal anesthesia for CS (Figure 2). The results of the survey conducted among anesthesiologists who performed GA for CS during the study period are given in Table 2.

**Table 1:** Demographic data, ASA status, reasons for exclusion, and type of surgery based on urgency of all patients and those with DA. DA, definite awareness; ASA, American Society of Anesthesiologists; CS, Caesarean section

Variables	All patients *n *= 1,161	Patients with DA, *n *= 5
Age (years)	31.28 (SD 5.29)	31.4 (SD 3.87)
Education, *n* (%)		
Without formal education	6 (0.5%)	-
Elementary school	42 (3.6%)	-
High school education	805 (69.3%)	4 (80%)
Higher professional education	542 (46.6%)	1 (20%)
Postgraduate education	13 (1.1%)	-
ASA score		
2	1,148 (98.9%)	5 (100%)
3	13 (1.1%)	-
Type of surgery based on urgency, *n* (%)		
Elective CS	427 (36.7%)	3 (60%)
Emergency CS	734 (63.3%)	2 (40%)
Patients with chronic illnesses	291 (25.1%)	1 (20%)
Patients with chronic therapy	223 (19.2%)	1 (20%)
Reasons for excluding patients from the study, *n *= 75		
Severe peripartum bleeding	20 (26.6%)	
Postpartum convulsions	2 (2.6%)	
Inadequatly completed questionnaries	20 (26.6%)	
Subsequent refusal to participate in the study	25 (33.3%)	
Death of the newborn	8 (10.6%)	
Professional experience of the anesthesiologists, *n* (%)		
Senior anesthesiologists	23 (71.9%)	
Residents	9 (28.1%)	
˂10 years of professional experience	17 (53.1%)	

 

**Table 2:** Survey results conducted among anesthesiologists performing anesthesia for CS in all medical centers. *Some of the senior anesthesiologists participating in the survey were residents at the time the study was conducted. ∞In some hospitals, senior anesthesiologists are present during induction and emergence and the rest of the operation is managed by the residents. CS, Caesarean section

Questions	Answers	*n* (%)
Senior anesthesiologists or residents	Senior	23 (71.8)
	Residents	9 (28.2)*
Residents performed anesthesia on their own∞	Yes	16 (100)*
	No	0
Professional experience	˂10 years	17 (53.1)
	10-20 years	9 (28.1)
	˃20 years	6 (18.7)
Intravenous anesthetic most commonly used during induction	Propofol	19 (59.4)
	Thiopental	13 (40.6)
Neuromuscular relaxant most commonly used during induction	Succinylcholine	32 (100)
Inhalational anesthetic for maintaining anesthesia	Sevoflurane	32 (100)
Neuromuscular relaxation for maintaining anesthesia	Always	28 (87.5)
	Often	3 (9.4)
	Sometimes	1 (3.1)
	Never	0
Neuromuscular relaxant most commonly used during induction	Atracurium	28 (87.5)
	Pancuronium	4 (12.5)
Use of nitrous oxide	Always	17 (53.1)
	Often	8 (25)
	Sometimes	4 (12.5)
	Never	3 (9.37)
Nitrous oxide : oxygen	50:50	22 (68.8)
	60:40	7 (21.8)
Turning off inhalational anesthetic during fetal extraction	Always	12 (37.5)
	Often	8 (25)
	Sometimes	8 (25)
	Never	4 (12.5)
Weaning off inhalational anesthetic at the end of operation	Immediately before placing stiches on the skin	13 (40.6)
	During the placement of stitches on the skin	14 (43.75)
	After covering operative wound	3 (9.4)
Premedication before CS	Always	1 (3.12)
	Often	1 (3.12)
	Sometimes	7 (21.8)
	Never	23 (71.8)

A total of 12 patients (1.03%) from all five centers reported that they had memories in the period between induction and anesthesia emergence (AE). In consultation with clinical psychologists who examined patients suspected of AAGA, it was determined that five of them had definite AAGA (0.43%) and the remaining seven (0.60%) had possible or unlikely awareness.

The average age of all patients who reported possible awareness was 31.4 (95% confidence interval [CI] SD ±3.801 (±12.10%)). According to MACI in patients with definite awareness, two patients had tactile sensations, one patient had a feeling of paralysis, and two felt pain. Patients with the sensation of pain report significant psychological trauma.

Patients with definite awareness gave detailed explanations that were characterized as definite awareness (Table 3). Out of five, three patients had experienced at the very end of the operation, as a result of premature emergence from anesthesia, where they woke up at the moment of placing stitches on the skin. Of these three, two felt pain and highlighted significant psychological trauma and stress. We identify seven other patients as possible or unlikely AAGA (Table 4).

**Table 3:** Patients with definite awareness. Description of intraoperative memories of the patients with definite awareness. MACI, Michigan Awareness Classification Instrument

Description of awareness	Operation/anesthesia phase	MACI2
Patient heard people around her talking and laughing, had the feeling that she wanted to open her eyes but was not able to, and felt stitches being placed on her skin. The patient did not perceive the events as trauma, and she did not feel any pain.	Anesthesia emergence	Class 4
The patient claimed that after she fell asleep, she woke up for a short period of time with a feeling of rocking and tearing in her stomach and awareness lasted for a short time; she did not perceive it as a traumatic experience; she did not feel pain; and there was no anxiety.	Immediately after anesthesia induction	Class 2
The patient stated that she woke up for a moment during the procedure, and she felt that she had a tube in her mouth, and soon after, she fell asleep again.	During operation	Class 2
The patient remembered the pain when the stitches were placed at the end of the operation.	Anesthesia emergence	Class 3D
The patient remembered the pain when the stitches were placed at the end of the operation.	Anesthesia emergence	Class 3D

**Table 4:** Patients with possible and unlikely awareness. Each of these patients filled in the questionnaire by themselves during two time intervals without talking to a researcher. All patients marked these answers in the first MCQ questionnaire given 24 hours after the surgical procedure, but in the second questionnaire, 72 hours later, they marked other offered answers. There were no descriptions of the memories in the questionnaires, only marked answers in MCQs. Given the inconsistency in responses across two different time periods, we did not define these responses as definitive awareness. MACI, Michigan Awareness Classification Instrument; MCQ, multiple-choice question

Description of memories	MACI
The patient stated that she heard voices between falling asleep and waking up.	Class 1
The patient stated that she heard voices between falling asleep and waking up.	Class 1
The patient stated that she heard voices between falling asleep and waking up.	Class 1
The patient stated that she heard voices and felt that she was being operated on but did not feel any pain.	Class 2
The patient felt that she was being operated on but did not feel any pain.	Class 2
The patient stated that she was awake and could not move or breathe.	Class 4
The patient felt pain during the operation.	Class 3

**Figure 3:**
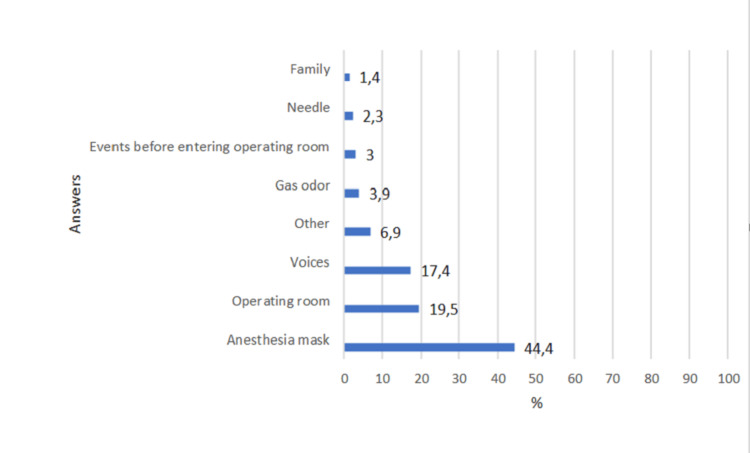
Distribution of answers to the following question: What is the last thing you remember before falling asleep?

**Figure 4:**
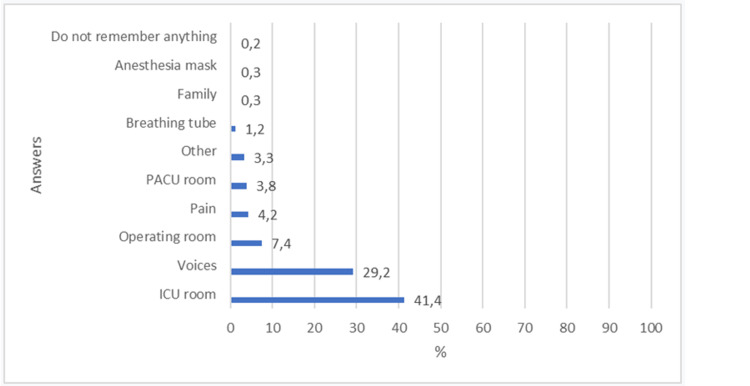
Distribution of answers to the following question: What is the first thing you remember after waking up?

**Figure 5:**
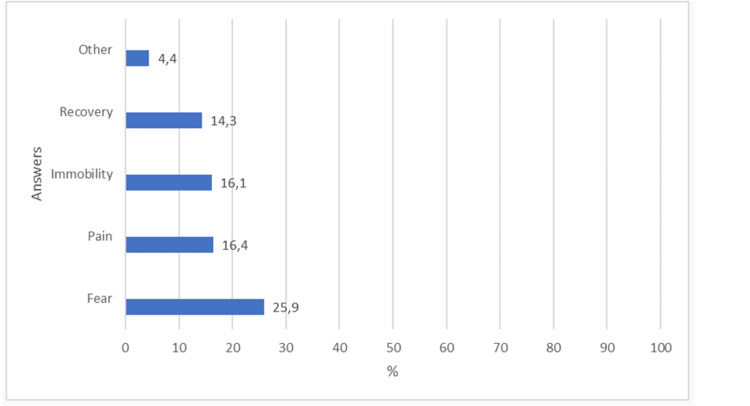
Distribution of answers to the following question: What was the worst thing about the operation?

Nineteen patients reported that they remember the presence of the tube and waking up from anesthesia. These reports were not characterized as intraoperative awareness. The distribution of answers to the questions in the Brice questionnaire is shown in Figures 3-5.

Intraoperative dreaming was also examined. Out of 42 patients (3.61%) who reported having dreams during the operative procedure, five (0.43%) stated that the dreams were unpleasant and two gave descriptions that could indicate intraoperative awareness. Analyzing the statements of these two patients, no connection between their dreams and intraoperative awareness was observed.

## Discussion

The study aimed to evaluate the incidence of AAGA and dreaming in patients undergoing CS under GA in five medical centers in RS (BIH). Of the 1,161 patients included in the study, five had definite awareness, which represented the AAGA incidence of 0.43%. Compared to the reported incidence from earlier research [9,12,13], this finding is considered significant. More than 50% of the patients in this study had AAGA at the end of the operation, in the phase of AE. The Fifth National Audit Project (NAP5) study finds that about 18% of patients reported remembering the endotracheal tube or not being able to move during AE [9]. In a review article on AAGA at the end of the surgery, that is, during emergence from anesthesia, Cascella et al. pointed out that according to previous studies, this type of awareness amounted to about 20% of all patients reporting awareness. At the same time, the authors pointed out that this type of awareness was extremely important because it represented an extremely stressful event for the patient and can cause various psychological disorders in the postoperative period [14].

Possible reasons explaining why a significant percentage of patients in this study reported awareness at the very end of the operative procedure are inexperienced personnel and shutting off the vaporizer too early in the process of AE.

We surveyed anesthesiologists from the medical centers where the research was conducted (Table 2). Thirty percent of them were residents, and more than 50% of all surveyed anesthesiologists had less than 10 years of experience. Inoue et al. conducted a study in which they investigated whether memories of the endotracheal tube (ET) and AE could be related to the lack of experience by the anesthesiologist and whether anesthesia administered by residents had a higher incidence of these adverse events [15]. This study found that 7% of the patients recalled ET and could not relate this finding to the lack of experience or administration of anesthesia by residents [15]. Forty percent of the surveyed anesthesiologists in our study shut off the vaporizer just before placing stitches. This leads to a certain number of patients reporting AAGA at the end of the surgery procedure.

Some studies reported ~50% of cases of awareness in the stages immediately after induction of anesthesia [9]. For CS, the usual practice in the operating room (OR) is to first clean and cover the surgical field and then perform anesthesia induction. The operative procedure begins right after checking the adequate ET position. If anesthesia is inducted this way, there is a period of light anesthesia in the moments when the effect of the intravenous anesthetic subsides before the inhalation anesthetic effect takes place. This is called an *intravenous-inhalation interval*. This period is considered the most susceptible to the occurrence of AAGA during CS [16,17]. One of the patients in this study with definite awareness described the experience of being suddenly awakened by a *rocking and tearing* feeling in her stomach after falling asleep for a short period. This experience could correspond to the moment of fetal extraction when the effect of the intravenous has subsided, and the effect of the inhaled anesthetic has not yet occurred (*intravenous-inhalation interval*). Only this patient reported AAGA in the phase immediately after induction of anesthesia. This number is lower compared to data from other studies [9,14] even though over 80% of our surveyed anesthesiologists always and often shut off the vaporizer during the phase of fetal extraction, and 20% of them never or rarely used nitrous oxide.

According to the survey results in this research, propofol was the choice for induction of anesthesia for CS by 60% of surveyed anesthesiologists in hospitals in RS. In general, thiopental is still used and preferred for the induction of anesthesia during CS due to its more favorable properties in terms of faster onset of action, lower incidence of awareness, less pronounced hypotension, and better Apgar scores in newborns [17]. However, the use of thiopental has decreased significantly. Propofol is an adequate substitute for thiopental and more studies support the use of propofol in obstetrics [18,19]. According to some studies, the use of thiopental was associated with a greater risk for the occurrence of AAGA [9]. In this study, propofol was used in around 60% of cases and this could be the reason for reduced awareness in the phases during and immediately after the anesthesia induction. This observation requires further research to prove it.

The study has certain limitations and potential sources of error. *Recall bias* is possible as the interpretation of patients' memories may be wrong. Some patients were interviewed in a direct conversation with the researcher, while others filled in the questionnaires by themselves. During the conversation, the influence of the researcher on the choice of answers could be possible. If patients filled in the questionnaire by themselves, it could be possible that they did not understand the questions and consequently gave wrong answers. In certain hospitals, not all patients who underwent CS were included in a study due to the lack of researchers. For the same reason, most of the patients were interviewed only 24 hours after the operation, and only those with suspected AAGA were interviewed once again after 72 hours. We did not have appropriate conditions to interview patients at least once again 30 days after the operation. All of the mentioned could lead to possible missed cases of awareness. The study is observational, research conditions were not strictly controlled. Anesthesia is managed according to the personal choices and preferences of the anesthesiologists. Anesthesia reports have not been analyzed and we did not make exact correlations between anesthesia management and cases of awareness.

## Conclusions

The incidence of AAGA during caesarean section in medical centers in RS (BIH) is 0.43%. This incidence is significant and is higher compared to earlier studies. During CS AAGA can appear at different stages of the operation and the most vulnerable phase is after anesthesia induction during the *intravenous-inhalation interval*. In our study, most of the patients reported awareness at the end of the procedure. These cases of AAGA are usually preventable. In this research, we concluded that the lack of clear anesthesia management protocols for CS and the lack of experience of anesthesiologists who perform anesthesia contribute the most to this finding. More research under better-controlled conditions is necessary to understand the true nature of this rare anesthesia complication.
